# Exploring traditional medicine utilisation during antenatal care among women in Bulilima District of Plumtree in Zimbabwe

**DOI:** 10.1038/s41598-021-86282-3

**Published:** 2021-03-25

**Authors:** Nicholas Mudonhi, Wilfred Njabulo Nunu, Nomathemba Sibanda, Nkosana Khumalo

**Affiliations:** 1 Department of Environmental Science and Health, Faculty of Applied Sciences, National University of Science and Technology, Corner Gwanda Road and Cecil Avenue, P O Box AC 939, Ascot, Bulawayo, Zimbabwe; 2 Scientific Agriculture and Environment Development Institute, Bulawayo, Zimbabwe

**Keywords:** Environmental social sciences, Health care, Medical research

## Abstract

Traditional medicine utilisation during antenatal care has been on the increase in several countries. Therefore, addressing and reinforcing the Sustainable Development Goal of maternal mortality reduction, there is a need to take traditional medicine utilisation during pregnancy into consideration. This paper explores traditional medicine utilisation during antenatal care among women in Bulilima District of Plumtree in Zimbabwe. A cross-sectional survey was conducted on 177 randomly selected women using a semi-structured questionnaire. Fisher's Exact Test, Odds Ratios, and Multiple Logistic Regression were utilised to determine any associations between different demographic characteristics and traditional medicine utilisation patterns using STATA SE Version 13. The prevalence of Traditional Medicine utilisation among pregnant women was estimated to be 28%. Most traditional remedies were used in the third trimester to quicken delivery. The majority of women used holy water and unknown Traditional Medicine during pregnancy. There was a strong association between age and Traditional Medicine utilisation as older women are 13 times more likely to use Traditional Medicine than younger ones. Women use traditional medicine for different purposes during pregnancy, and older women's likelihood to use Traditional Medicine is higher than their counterparts. The traditional system plays an essential role in antenatal care; therefore, there is a need to conduct further studies on the efficacy and safety of utilising Traditional Medicines.

## Introduction

Maternal health is generally of global concern, and to ensure safe pregnancies and delivery, several countries have been challenged to provide adequate maternal and child health services as enshrined on the Sustainable Development Goal (SDG) of reduction in maternal mortality of 7.5% per year between 2016 and 2030^1–3^. However, different countries utilise different health systems to achieve these global targets^4,5^. In Africa (particularly in Sub-Saharan Africa), access to modern health facilities is a challenge due to exorbitant costs associated with it and the health care recipients' economic status^6^.

Traditional medicine (TM) utilisation has been on the upsurge in several African countries as it plays a vital role during antenatal care^7^. It could contribute positively or negatively towards the attainment of SDG 3, emphasising reducing the Global Maternity mortality rate to 70 per 100,000^3,7,8^. The use of traditional medicines in pregnancy management induces and shortens labour is a well-established practice among African countries^9,10^. Reported reasons for TM utilisation during pregnancy include; promotion of foetal growth, spiritual cleansing, protection against evil influence, to have a male child and assisting childbirth just to mention a few^11,12^. The route of TM exposure during antenatal care varies; some are ingested, inhaled, or applied as an ointment for different purposes^13,14^. Determinates such as women's belief, lower cost, and accessibility of TM triggers them to have trust in their effectiveness compared to western medicines^15^.

In the Zimbabwean context, preference to deliver at home and utilisation of TMs has been influenced by the cost of health care, distance, educational level, and religion^16–18^. Traditional medicines have been utilised since the pre-colonial era, with over 80% of the population still relying on traditional remedies and the Indigenous Knowledge (IK) being passed down to generations^19,20^. Women prefer birth attendants that understand their spiritual background, and they feel at peace when they perform their cultural activities that are believed to be beyond human capabilities^17^. In addition, the Zimbabwe Maternal and Perinatal Mortality Study conducted by the Ministry of Health and Child Care in 2007 found that women prefer to go into labour at traditional birth attendants and faith healers' homes^21^. In Zimbabwe's rural areas, lack of access to western medicines has been an influencing factor for women to use TM. In addition, better modern health facilities with required expertise and equipment are largely centralised in urban setups, making it difficult for rural women to access these services^22^.

There have been several strategies that have been implemented in rural Zimbabwe including the establishment of Maternal Waiting Homes (MWH) to try and reduce barriers such as cost of transport, distance and prevent maternal complications (just to name a few) to improve access of women to modern maternal health services^3,23^. However, most women still prefer to utilise TM despite campaigns that discourage women from utilising TM as some have unforeseen adverse reactions^24^. The majority of users; therefore utilise TM secretly and rarely disclose to the health service providers. In Plumtree, particularly in Bulilima District, the average distance walked by women to the nearest health facility is estimated at between 5 and 10 km, influencing them to consult the traditional system which is readily available in their communities^25^. Generally, it is suggested that there should be a health facility within a 5 km radius in different communities and women should not walk more than the 5kms in search of maternal services^18^. Therefore, this study explores traditional medicine utilisation trends during antenatal care among women in Bulilima District of Plumtree in Zimbabwe. This study presents a window of opportunity to determine the TM utilisation patterns that would inform policy makers in coming up with strategies that would strengthen the current existing health systems.

## Methods

### Study area

**Figure 1:**
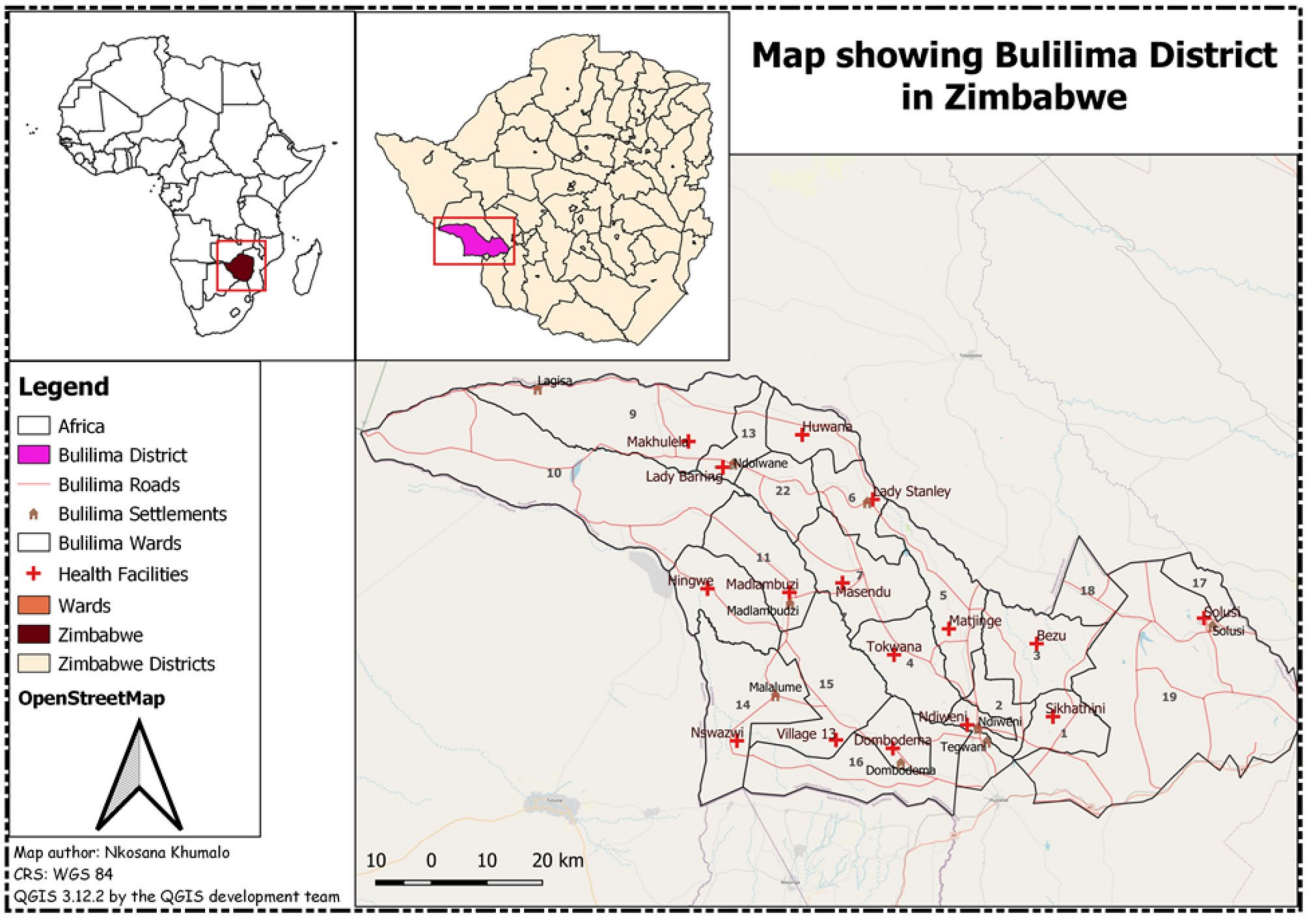
Map showing Bulilima District and its health facilities.

Bulilima is one of the seven districts with 22 wards located in Matabeleland South province and is in Region 5, prone to severe drought^26^. The district has one main referral hospital with sixteen clinics that usually refer pregnant women with complications to a district hospital and has an average household size of 5^27^. Generally, it is estimated that this district is home to 57,681^28^. The average distance that women walk to the nearest clinic is estimated to be 5–10 km. The study area is illustrated in Fig. 1 which was developed using Quantum Geographic Information System (Credit: QGIS 3.12.2 by the QGIS development team). Although a similar study was conducted in Harare, Zimbabwe which is an urban set up with a different population composition predominantly the Shona tribe, our study was entirely rural-based and in a different region of the country with predominantly Kalanga and Ndebele speaking people relying on different TM as compared to some other regions as the belief systems differ^27,29^.

### Study design

A cross-sectional survey that explored traditional medicine utilisation during antenatal care among women in Bulilima District was conducted. This study design was appropriate as it enabled the exploration of traditional medicine utilisation trends in a single point in time, ensuring cost-effectiveness as this study was not funded^30^.

### Target population

This study targeted all women who delivered from January-December 2019 (to minimise recall bias) in Bulilima District as captured in the health facilities' birth registers. The women who met the inclusion criteria were 586, and there was no age limit.

### Sampling

A sample size calculator on EPI INFO Version 7.2.2.6 was used to estimate the minimum sample size required for this study. A confidence level of 95%, Width of Confidence of 5%, and the expected value of attribute applied to the study population of 586 gave an estimated sample size of 185. Random numbers were then generated, and the 185 selected and followed up.

### Data collection tools

Pre-testing of a semi-structured questionnaire and data collection was done by the researchers who are all Trained Public Health Specialists between January 2020-February 2020 from women delivered at the clinic or home in Bulilima district but registered at the clinic. The questionnaire was categorised into two sections, that is: the first section delved on socio-demographic characteristics (age, race, ethnicity, education, marital status, parity). The second section comprised questions on the source, different types, reasons, and frequency of TM used. The questionnaire was developed in English and then translated to the local language that is "isi*Ndebele,*" which is mainly spoken and taught within the district.

### Data analysis

Collected data was coded and entered into EpiData 3.1 then further exported to Microsoft Excel 2013. The analysis was done with the aid of STATA version 13; for instance, descriptive statistics were used for women's demographic characteristics. Fisher's Exact, Odds Ratios (OR), and Multiple Logistic Regression (MLR) were used to determine the presence and strength of associations between demographics and TM utilisation.

### Ethical approval and consent to participate

Permission to carry out the study was sought from relevant authorities that are Provincial Medical Director for Matabeleland South, District Medical Officer for Bulilima and National University of Science and Technology, particularly the Department of Environmental Science and Health. Moreover, the research abides by the Nuremberg code and principles stated in the Helsinki Declaration for the safety of participants involved in the study^46^. Written consent was obtained from all the respondents who participated in the study. Permission was sought from parents of adolescents who were less than 18 years of age, and there were also required to assent to the study.

## Results

### Response rate and demographics characteristics of women

Out of the targeted 185 women, 177 responded to a pre-tested questionnaire presenting a response rate of 96%. Some of the women had left their places of residence and could not be obtained. However, a response rate of 96% was considered sufficient by the researchers to make meaningful inferences. The majority of women were having a partner 132 (74.6%), and 139 (78.5%) are Christians, while 110 (62.2%) are unemployed. Also, the results show that only one woman was within the age of 50–54, as indicated in Table 1:

**Table 1:** Demographic Characteristics of respondents and Traditional Medicine Utilisation.

Traditional medicines utilisation								
Variable	Didn't use TM		Used TM		Fisher Exact	MLR-OR	MLR-95% CI	MLR P-value
	Freq	%	Freq	%				
**Age**								
15–19*	33	26.0	5	10.0	0.011	***		
20–24	26	20.5	7	14.0		1.78	0.51–6.25	0.37
25–29	25	19.7	9	18.0		2.38	0.71–7.97	0.16
30–34	18	14.2	8	16.0		2.93	0.84–10.30	0.09
35–39	18	14.2	9	18.0		3.30	0.96–11.35	0.06
40–44	5	3.9	7	14.0		9.24	2.10–40.75	< 0.01
45–49	2	1.6	4	8.0		13.2	1.90–91.91	< 0.01
50–54	0.00	0.0	1	1.0		1		
Mean (sd) 29.1 years (9.1)	27.4 (8.4)	33.4 (9.6)						
**Marital status**								
Single*	18	14.2	7	14.0	0.853	***		
In a relationship	45	35.4	14	28.0		0.80	0.28–2.31	0.68
Married	42	33.1	18	36.0		1.10	0.39–3.10	0.85
Widowed	7	5.5	5	10.0		1.84	0.43–7.77	0.41
Divorced	6	4.7	2	4.0		0.86	0.14–5.31	0.87
Cohabiting	9	7.1	4	8.0		1.14	0.26–4.95	0.86
**Tribe**								
Ndebele***	58	45.7	18	36.0	0.349	***		
Shona	14	11.0	4	8.0		0.92	0.27–3.15	0.90
Kalanga	50	39.4	24	48.0		1.55	0.75–3.17	0.23
Tonga	3	2.4	1	2.0		1.07	0.11–10.97	0.95
Other	2	1.6	3	6.0		4.83	0.75–31.23	0.10
**Religion**								
Christian***	104	81.9	35	70.0	0.002	***		
Traditional	11	8.7	14	28.0		3.78	1.57–9.10	< 0.01
None	12	9.5	1	2.0		0.25	0.03–1.97	0.19
**Level of education**								
Primary***	35	27.6	13	26.0	0.342	***		
Ordinary level	54	42.5	24	48.0		1.20	0.54–2.66	0.66
Advanced level	23	18.1	8	16.0		0.94	0.34–2.61	0.90
Tertiary	11	8.7	1	2.0		0.24	0.03–2.09	0.20
Never attended school	4	3.2	4	8.0		2.69	0.59–12.37	0.20
**Employment status**								
Employed	22	17.3	12	24.0	0.495	***		
Self Employed	23	18.1	10	20.0		0.80	0.29–2.22	0.66
Unemployed	82	64.6	28	56.0		0.63	0.27–1.43	0.26
**Place of delivery**								
Hospital	99	78.0	32	64.0	0.086	***		
Home	28	22.1	18	36.0		1.99	0.97–4.06	0.06
**First child**								
Yes	47	37.0	6	12.0	0.001	***		
No	80	63.0	44	88.0		4.46	1.77–11.24	< 0.01
**Parity**								
1	48	37.8	6	12.0	0.002	***		
2–5	75	59.1	39	78.0		4.16	1.64–10.57	< 0.01
6 > =	4	3.2	5	10.0		10.00	2.09–47.82	< 0.01

Relationship between parity and age								
	Mean (sd)	Used TM (Mean; sd)	Did Not Use TM (Mean; sd)					
15–19	1.1 (0.3)							
20–24	1.5 (0.5)							
25–29	1.9 (1.3)							
30–34	2.0 (0.3)							
35–39	2.0 (0.2)							
40–44	2.7 (1.6)							
45–49	2.7 (1.6)							
50–54	2.7 (1.6)							
Overall	1.90 (1.1)	2.3 (1.3)	1.7 (0.9)					

### Traditional medicine used during antenatal

**Figure 2:**
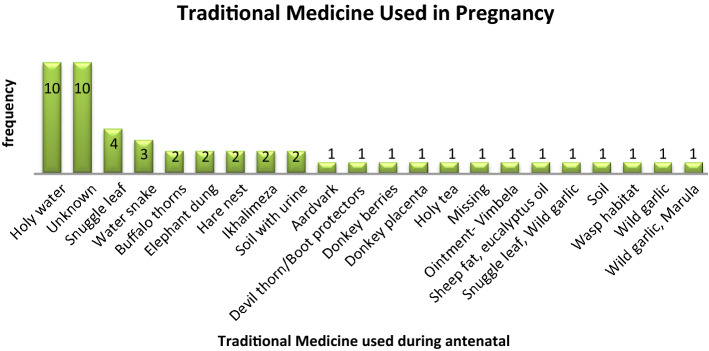
Traditional medicine used during antenatal (n = 50).

The majority of individuals used holy water and an unknown type of traditional medicine, while ten women used only one type of traditional medicine. Fig. 2 and Table 2 show different types of TMs used by women.

**Table 2:** Traditional medicine used.

Local name	Common name	Scientific name	Reasons for use	How it is used	Trimesters
Isikhukhukhu	Snuggle-leaf	*Pouzolzia hypoleuca* Wedd	Fast delivery	Mix with water and drink	3rd
Inkunzane	Boot protectors/Devil thon	*Dicerocaryum species*	Lubricate the birth canal	washing the birth canal	3rd
umphafa	Buffalo thorn	*Ziziphus mucronata*	Manage breech	Drinking	1,2 and 3
Ubhuzu	Donkey Berry	*Grewia flavescens*	Manage breech	mix its roots with water and drink	1,2 and 3
Umganu	Marula	*Sclerocarya caffra*	Inyongo (fever)	Drinking	1st
Isihaqa	Long tail Cassia Wild garlic	*Cassia abbreviate*	Stomach pains	Roots/barks mix with water and drink	1st
Ikhalimela			For fast delivery	Mix with cold water and drink	3rd
Nyeluka	Water snake/fish		Fast delivery	Mix its skin with water and drink	3rd
Inqwatshi kababhemi	Donkey Placenta		Fast delivery	Mix with water and drink	3rd
Ubulongwe bendlovu	Elephant Dung		Manage bleeding	Fumigating	1,2 and 3
Ivimbela	White/Red ointment		Chase evil spirits and manage breech	Mix with Vaseline and massage stomach	1,2 and 3
Ifutha lemvu	Sheep Fat		Manage breech and protect from witchcraft	Fumigating and anointing	1,2 and 3
	Eucalyptus oil		Protect the baby from witchcraft	Anointing, mix with water and drinking	1,2 and 3
Muzemuze	Wasp Habitant		Fast delivery	Take its habitat mix with water and drink	3rd
Isikhundla sikamvundla	Hare nest		Fast delivery	Mix its soil with water and drink	3rd
Amanzi Angcwele	Holy water		Protect from evil spirits and witchcraft	Drinking and bathing	1,2 and 3
Itiye Elingcwele	Holy tea		Protect from evil spirits and witchcraft	Drinking and bathing	1,2 and 3
Inhlabathi elomthambiso	Dried soil with urine		Prevent from tying and witchcraft	tie dried soil with urine in a cloth	1st and 3rd
Ukuchupha unyawo	Footprint soil		Prevent from tying and witchcraft	tie soil in a cloth	1st and 3rd
Ukuzinuka Amakhwapha	Smelling your armpit		Prevent from vomiting	Putting nose under armpit	anytime

### Prevalence and safety perception of traditional medicine use

The prevalence of TM use was 50 (28.3%) during pregnancy, and also a more significant number of women use traditional medicines during their third trimester. Table 3 clearly shows prevalence, safety, and other variables of traditional medicines utilisation pattern.

**Table 3:** Prevalence and safety perception of traditional medicine use.

TM prevalence (n=177)	Frequency	%		
Used TM	50	28.3		
Did not use TM	127	71.8		
**Views on safety**				
Safe	20	11.3		
Not Safe	39	22.0		
Don't Know	118	66.7		

Period & type of Traditional Medicine ↓	Frequency of traditional medicine use (n = 50)			Total
	1–5 times	6–10 times	11 >	
**1st trimester**				
Holy water	0	2	3	5
Buffalo thorns	2	0	0	2
Wild Garlic/Marula	2	0	0	2
Soil/Soil with urine	1	1	0	2
Unknown	1	0	1	2
Elephant dung	1	0	0	1
Missing	0	0	0	1
**2nd trimester**				
Holy water	0	2	3	5
Soil	0	1	0	1
Unknown	1	0	1	2
**3rd trimester**				
Aardvark	1	0	0	1
Devil thorn/Boot protector	0	0	1	1
Donkey berries	1	0	0	1
Elephant dung	1	0	0	1
Hare nest	2	0	0	2
Holy tea	0	1	0	1
Holy water	0	3	6	9
Ikhalimeza	0	2	0	2
Ointment-Vimbela	0	0	1	1
Sheep fat and eucalyptus oil	0	0	1	1
Snuggle leaf	2	0	0	2
Soil/ Soil with urine	1	1	0	2
Unknown	2	0	5	7
Wasp habitat	1	0	0	1
Water snake	2	1	0	3
**During labour**				
Donkey placenta	1	0	0	1
Holy water	1	0	0	1
Snuggle leaf	2	1	0	2
Soil with urine	1	0	0	1
After labour	1	0	0	1
Devil thorn/boot protectors	1	0	0	1
Unknown	1	0	0	1

### Demographic characteristics and TM use

There was a strong significant association between age and TM utilisation as older women are 13 times more likely to use TM than younger ones. Religion and parity were associated with TM use. On the other hand, marital status, Tribe, Level of education, employment status, and place of delivery was not associated with TM utilisation as shown in Table 1. Age is the only variable significantly associated with the frequency of TM use during pregnancy, as indicated in Table 4.

**Table 4:** Demographics and frequency of TM use.

Variable (n = 50)	1–5 times	6 >	Fisher`s exact	MLR-OR	MLR-95% CI	MLR P-value
**Age**						
15–19	0 (0.0)	5 (20.8)	0.026	1		***
20–24	4 (15.4)	3 (12.5)		2.25	0.15–34.00	0.59
25–29	4 (15.4)	5 (20.8)		3.75	0.27–51.37	0.99
30–34	4 (15.4)	4 (16.7)		3	0.21–42.62	0.81
35–39	4 (15.4)	5 (20.8)		3.75	0.27–51.37	0.99
40–44	7 (26.9)	0 (0.0)		1		
45–49	3 (11.5)	1 (4.2)		1		
50–54	0	1 (4.2)		1		
**Marital status**						
Single	1 (3.9)	6 (25.0)	0.057			***
In a relationship	5 (19.2)	9 (37.5)		0.30	0.28–3.25	0.322
Married	12 (46.2)	6 (25.0)		0.08	0.01–0.86	0.037
Widowed	4 (15.4)	1 (4.2)		0.04	0.01–0.88	0.041
Divorced	2 (7.7)	0 (0.0)		1		
Cohabiting	2 (7.7)	2 (8.3)		0.17	0.01–2.98	0.097
**Tribe**						
Ndebele	8 (30.7)	10 (41.7)	0.910	***		
Shona	2 (7.7)	2 (8.3)		0.80	0.09–7.00	0.840
Kalanga	13 (50.0)	11 (45.8)		0.68	0.20–2.31	0.534
Tonga	1 (3.9)	0 (0.0)		1	0.03–5.25	0.485
Other	2 (7.7)	1 (4.2)		0.40	0.49–3.18	0.638
**Religion**						
Christian	16 (61.5)	19 (79.2)	0.278	***		
Traditional	9 (34.6)	5 (20.8)		0.47	0.13–1.68	0.245
None	1 (3.9)	0 (0.0)		1		
**Level of education**						
Primary	5 (19.2)	8 (33.3)	0.308	***		
O**'**level	11 (42.3)	13 (54.2)		0.74	0.19–2.92	0.666
A`level	6 (23.1)	2 (8.3)		0.21	0.03–1.47	0.115
Tertiary	1 (3.9)	0 (0.0)		1		
Never attended school	3 (11.6)	1 (4.2)		0.21	0.02–2.60	0.223
**Employment status**						
Employed	4 (15.4)	8 (33.3)	0.237	***		
Self Employed	7 (26.9)	3 (12.5)		0.21	0.04–1.31	0.095
Unemployed	15 (57.7)	13 (54.2)		0.43	0.11–1.78	0.246
**Place of delivery**						
Hospital	15 (57.7)	17 (70.8)	0.388	***		
Home	11 (42.3)	7 (29.2)		0.56	0.17–1.82	0.336
**First child**						
Yes	3 (11.5)	3 (12.5)	1.000	***		
No	23 (88.5)	21 (87.5)		0.91	0.166–5.03	0.917
**Parity**						
1	3 (11.5)	3 (12.5)	0.515	***		
2–5	19 (73.1)	20 (83.3)		1.03	0.16–6.82	0.870
6 ≥	4 (15.4)	1 (4.2)		0.25	0.02–3.77	0.317

*** Reference group.

## Discussion

The study found out that most women had a partner, were Christians, and was unemployed. Most researchers that conducted studies in Zimbabwe supports our findings as they revealed that most women attending antenatal care in public institutions are unemployed and are in a relationship^31,32^. Results indicated that older women's likelihood of using traditional medicine during pregnancy is higher than their younger counterparts. These findings are supported by a study conducted in Taiwan, which indicates that older women are likely to use traditional, complementary medicines than their younger counterparts^33^. Findings denote that marital status, Tribe, Level of education, employment status, and place of delivery were not significantly associated with traditional medicine utilisation. Studies conducted in Zimbabwe concur with our findings that religion is not related to the use of TMs during pregnancy^34^.

Our findings indicated that the prevalence of TM use was 28.25%. Most scholars who conducted their studies on maternal health and traditional medicine use in Sub-Saharan countries (Zimbabwe 52%, Nigeria 68%, Mali 80%, South Africa 55–93.6%, Mali 80%, Tanzania 55%) contradicts with our findings as they note that the prevalence ranges from 52 to 80%^29,35–39^. Even though other scholars contradict our findings, multinational studies conducted in Europe, Australia, South, and North America are aligned with our results as they revealed a prevalence of 28.9% use herbal medicine during pregnancy^7^.

Women revealed in our study that they use several TMs to induce and shorten labour, these include isikhukhukhu (Snuggle-lea: *Pouzolzia hypoleuca* Wedd), and inkunzane (Boot protectors/devil thorn; *Dicerocaryum species*). Other scholars who conducted their studies in Zimbabwe concur with our results as they indicate that Snuggle-lea (*Pouzolzia hypoleuca* Wedd) was used to induce labour^9,11^. It is highlighted in this study that the majority of individuals were using holy water and an unknown type of traditional medicine. These results are in line with a study conducted by Mureyi^29^, that indicated holy water as a common TM used. In addition, scholars have noted that several herbs and their compounds are used during pregnancy are unknown^40–42^.

In Zimbabwe the Traditional health system is recognised and plays an important role in ensuring services are available to those that need them^43^. In pursuance of SDG (3), there is a need to ensure that utilization of traditional medicines leads to outcomes that do not jeopardise progress towards attaining this specific goal on maternal health^44,45^.

## Limitations

This study cannot be generalised to the entire country since the study population was rural-based and can be affected by recall bias even though women recruited gave birth during January-December 2019. Above all, the research was not funded, and as such, there could have been a need for a substantial cohort to make meaningful inferences. Authors are also involved in a project that intends to explore maternal complications and TM use and find the active ingredient of TM used by women during antenatal care.

## Conclusion

Women indeed used traditional medicine for different purposes during pregnancy, and the likelihood of older women to use traditional medicines was higher than in young women. Most dominant traditional remedies were used in the last trimester to quicken delivery by women. TM utilisation plays a significant role in pregnancy; therefore, there is a need that particular attention is paid to it and possibly more research to be conducted to assess its efficacy, safety as it gives a cheaper alternative to women who might not afford to access conventional modern health services.
